# 

**Published:** 1898-05

**Authors:** 


### MARRIAGES.

Dr. George P. Biggs, Secretary of the New York College of Veterinary Surgeons, and professor of physiology, was married April 14th, at the Fifth Avenue Presbyterian Church, New York City, to Miss Florence Browning, of New York. Dr. Herman M. Biggs was best man. The bride was attended by six bridesmaids and a maid of honor.

Miss Willard, daughter of W. A. Willard, who for a number of years has been in the employ of the Bureau of Animal Industry, was married in the First Presbyterian Church of Passaic, to Edward W. Berry, manager of the Passaic *News.* The wedding was a very elaborate affair; every seat in the church was occupied.

---

The action of the members of the Veterinary Medical Society of students of the University of Pennsylvania, in tendering their services in connection with the cavalry service of the United States for the war with Spain, was acknowledged on behalf of the President with appreciation of the spirit of the services tendered.

### PERSONALS.

Dr. M. P. Ravenel was a lecturer at the Farmers' Institute at Huntingdon, Pa., in March.

Dr. A. S. Wheeler, of Biltmore, N. C., has entered the Orthopedic Hospital of Philadelphia for a course of treatment.

Dr. M. J. Collins, of Myerstown, Pa., was on the sick list early in March.

Dr. D. E. Salmon, of the Bureau of Animal Industry, made a trip to Texas early in March to investigate the method of dipping cattle for extermination of ticks.

Dr. Louis A. Klein, Veterinary Department, University of Pennsylvania, Class of 1897, who recently accepted the position as veterinarian of Biltmore, Mr. Vanderbilt's estate in North Carolina, has resigned to accept a position as Federal meat-inspector in Chicago. Dr. Klein is succeeded at Biltmore by his classmate, Dr. James Beatty, of Philadelphia.

Dr. John W. Eshleman, of Parkesburg, Pa., has been selected as one of the ground committee of the Gentlemen's Driving Association.

Veterinarian E. C. Porter, of Newcastle, Lawrence County, Pa., won the Republican nomination for coroner of that county in March. Three M.D.'s were also in the race. All the candidates were raised there, except Dr. Porter, and, consequently, he had not only the candidates, but all their relatives to fight. Dr. E. E. Bittles, of New Castle, was a strong ally of Dr. Porter in the fight.

Dr. J. L. Tyler, of Chebanse, Ill., has abandoned the practice of veterinary medicine, and will locate at Jackson, Miss., where he will engage in the practice of human medicine.

Dr. Lloyd Zaner, of Stillwater, Pa., was a visitor to Philadelphia and the JOURNAL office in April.

Dr. Charles H. Canfield, graduate of the Ohio State University, and Dr. H. B. Chaney, graduate of the Ontario Veterinary College, both of Akron, Ohio, have been appointed meat-inspectors at Kansas City, Mo.

Dr. H. M. Burgess, of Pawtucket, R. I., has accepted an appointment in the Bureau of Animal Industry inspection-service, and assigned for duty at St. Joseph, Mo.

Dr. Charles T. Goentner, of Bryn Mawr, Pa., received a painful injury in April by the passage of a chisel through his hand.

Dr. J. C. McNeil, of Pittsburg, Pa., was confined to his bed for a week in April with a severe attack of lumbago.

Dr. A. W. Bitting, of Lafayette, Ind., was a recent contributor to the Jersey *Bulletin*, taking issue with Editor Jenkins upon his position relative to the great need of sanitary measures in the dairies.

Dr. H. J. McClellan, of Bryn Mawr, Pa., received the degree of M.D. at the Baltimore University School of Medicine, on April 17th. Dr. McClellan filled the rôle of President of his Class, and graduated at the American Veterinary College, Class of 1893.

Dr. Leroy M. Land, of Lexington, Ky., graduate of the Veterinary Department of the University of Pennsylvania, after a sojourn of two years in Germany, spent in advanced studies and laboratory work, has returned home, and will at once enter upon practice in the "Blue Grass State."

Dean Leonard Pearson, of the Veterinary Department of the University of Pennsylvania, was a guest of Vice-Provost Fullerton of that institution at a farewell dinner given prior to the latter's leave of absence for a year in Europe.

Dr. Joseph M. Good, of Chattanooga, Tenn., had the degree of M.D. conferred upon him by the Chattanooga Medical College on March 22d.

Dr. M. H. Reynolds, of Minnesota, addressed a meeting of farmers at Albert Lea on March 29th, on the "State-control of Hog-cholera," and will spend considerable time with the Minnesota State Farmers' Institute in the same work during the summer.

The Minnesota plan of having a field-veterinarian in the veterinary Department of the State Board of Health is proving very satisfactory. Dr. S. D. Brimhall, who has done this work for the Minnesota Board since the adoption of the plan, is almost constantly on the road and is of great assistance to local health-officers and owners of live-stock.

Dr. R. P. Steddom, formerly of Kansas City, has been transferred from that point to take charge of the quarantine service at Sacramento, Cal.

Dr. G. A. Johnson, of the Bureau of Animal Industry, has been transferred from Kansas City, Mo., to Sioux City, Ia.

Dr. James W. Sallade, of Pottsville, Pa., spent a week, in April, around Washington, Norfolk, and Hampton Roads.

Dr. E. P. Schaffter, formerly of Kansas City, is now located at the Cleveland (O.) department of the inspection service of the Bureau of Animal Industry.

Drs. Leonard Pearson, State Veterinarian, and M. P. Ravenel, Bacteriologist of the State Live-stock Sanitary Board, have been elected directors of the Society for the Suppression of Tuberculosis in Pennsylvania.

John J. Repp, of the Class of '98, veterinary department, represented the school at the arbor-day exercises of the University of Pennsylvania, in April.

Among those at the dispersal sale of the Cloverdale breeding-farm were to be noted Drs. H. D. Gill, of New York; H. P. Eves, of Wilmington, Del.; S. D. Larzelere, Jenkintown; C. H. Magill, Charles Williams, and Leonard Pearson, of Philadelphia; H. P. Turner, of Lahaska; J. Z. Hillegass, of Red Hill; and J. H. Oyler, of Harrisburg. Mr. Fasig said this was one of the best sales ever held in this country.

Dr. E. Schirmer, of Asheville, N. C., has removed to Washington, D. C.

Dr. W. G. Benner, of Doylestown, Pa., has proffered his services to Governor Hastings as veterinarian to a company of cavalry should his services be desired.

Dr. A. W. Clement, of Baltimore, will act as veterinarian to the annual horse-show of the Elk Ridge Fox-hunting Club, May 19th to 21st, inclusive.

Dr. J. A. Mowbray, of Oshawa, Ontario, was appointed Government inspector for South Ontario, in April.

Dr. W. W. Martin, Jr., of Philadelphia, has enlisted for two years and gone to the field with "Battery A," of Pennsylvania. Dr. Martin will fill the post of veterinarian to this company of artillery.

The Honorable Secretary of Agriculture has detailed Dr. C. W. Stiles, Zoölogist of the Bureau of Animal Industry, as Agricultural and Scientific Attaché to the United States Embassy at Berlin, Germany. Dr. Stiles leaves Washington immediately to assume his new duties. He still retains his official connection with the Bureau, and will eventually return to continue his investigations upon animal parasites.

Dr. E. C. Porter, of New Castle, Pa., is a candidate for coroner in his district.

Dr. Maurice O'Connell, a member of the Massachusetts Cattle Commission, succeeded Dr. J. J. Monyhan as City Veterinarian of Holyoke, Mass., on March 10th. The position carries with it a salary of six hundred dollars per year. Dr. O'Connell has for several years had full charge of the inspection, control, and eradication of glanders in the Bay State.

Dr. W. H. Ridge, of Trevose, Pa., bought "Orphant May," out of "Gypsy May," by "Forrest Wilkes," three years old, at McFarland's sale; colt owned by Col. Edward Morrell.

Dr. U. G. Houck is now in charge of the government meat-inspection service at Sioux City, Ia.

Dr. R. A. Hasbrouck, of Passaic, N. J., buried a son, twenty-four years of age, early in March, from tetanus following a runaway accident.

Dr. W. B. E. Miller, of Garfield, N. J., was a victim of rheumatism in February, and was confined to his bed for several weeks.

Dr. T. Earle Budd, of Woodbury, N. J., has been nominated for Council in that town.

Dr. H. P. Eves, of Wilmington, Del., has been on the sick list for the past month.

Dr. D. Fisher, of Grandin, North Dakota, combines the purchase and sale of horses with his practice.

Dr. H. W. Stedman, formerly of Toronto, Can., has located at Springfield, Mass.

Dr. Harry W. Turner, of Lahaska, Pa., was a visitor to the JOURNAL office recently.

Dr. E. L. Kellner sings in the choir of the Church of the Epiphany, Philadelphia.

Dr. William Dougherty, of Baltimore, Md., has become the proprietor of the Hotel Studio of the "Oriole City," where he will greet as host his many friends.

Dr. L. H. Howard, of Boston, suffered from a severe attack of conjunctivitis in March.

Drs. John T. Unertl and R. A. Higgins, of Wisconsin, are to be tried by their State association for violation of the code of ethics in connecting themselves with a livestock insurance company granting free treatment to owners of stock so insured.

The Directors of the Philadelphia Horse-Show Association have selected the following veterinarians to serve at the open-air show to be held during the last week in May: Drs. A. W. Clement, Baltimore, Md.; C. J. Marshall, Philadelphia, Pa., and W. H. Ridge, Trevose, Pa.

Dr. John A. Myers, of West Virginia, was a contributor to the February number of the *Southern Farm.*

